# Progenitor-like cells derived from mouse kidney protect against renal fibrosis in a remnant kidney model via decreased endothelial mesenchymal transition

**DOI:** 10.1186/s13287-015-0241-8

**Published:** 2015-12-02

**Authors:** CL Chen, KJ Chou, HC Fang, CY Hsu, WC Huang, CW Huang, CK Huang, HY Chen, PT Lee

**Affiliations:** Division of Nephrology, Department of Medicine, Kaohsiung Veterans General Hospital, School of Medicine, National Yang-Ming University, 386 Ta-Chung 1st Rd, Kaohsiung, 813 Taiwan

**Keywords:** Adult stem cells, Fibrosis, Chronic renal insufficiency, Cell-based therapy, Angiogenesis

## Abstract

**Introduction:**

Pathophysiological changes associated with chronic kidney disease impair angiogenic processes and increase renal fibrosis. Progenitor-like cells derived from adult kidney have been previously used to promote regeneration in acute kidney injury, even though it remained unclear whether the cells could be beneficial in chronic kidney disease (CKD).

**Methods:**

In this study, we established a CKD model by five-sixths nephrectomy and mouse kidney progenitor-like cells (MKPCs) were intravenously administered weekly for 5 weeks after establishing CKD. We examined the impact of MKPCs on the progression of renal fibrosis and the potential of MKPCs to preserve the angiogenic process and prevent endothelial mesenchymal transition in vivo and in vitro.

**Results:**

Our results demonstrate that the MKPCs delayed interstitial fibrosis and the progression of glomerular sclerosis and ameliorated the decline of kidney function. At 17 weeks, the treated mice exhibited lower blood pressures, higher hematocrit levels, and larger kidney sizes than the control mice. In addition, the MKPC treatment prolonged the survival of the mice with chronic kidney injuries. We observed a decreased recruitment of macrophages and myofibroblasts in the interstitium and the increased tubular proliferation. Notably, MKPC both decreased the level of vascular rarefaction and prevented endothelial mesenchymal transition (EndoMT) in the remnant kidneys. Moreover, the conditioned medium from the MKPCs ameliorated endothelial cell death under hypoxic culture conditions and prevented TGF-β-induced EndoMT through downregulation of phosphorylated Smad 3 in vitro.

**Conclusions:**

MKPCs may be a beneficial treatment for kidney diseases characterized by progressive renal fibrosis. The enhanced preservation of angiogenic processes following MKPC injections may be associated with decreased fibrosis in the remnant kidney. These findings provide further understanding of the mechanisms involved in these processes and will help develop new cell-based therapeutic strategies for regenerative medicine in renal fibrosis.

**Electronic supplementary material:**

The online version of this article (doi:10.1186/s13287-015-0241-8) contains supplementary material, which is available to authorized users.

## Introduction

Chronic kidney disease (CKD) is a global public health concern, and its burden continues to increase around the world, as demonstrated by increases in attributable deaths and the incidence of end-stage renal disease (ESRD) [1]. Regardless of the underlying causes, CKD features progressive loss of glomerular function associated with the presence of tubulointerstitial lesions and peritubular capillary rarefaction. At present, the therapeutic strategy for slowing the renal progression of CKD involves controlling blood pressure, using angiotensin-converting enzyme inhibitors or angiotensin II receptor blockers, and restricting protein [2]. The effects of these modalities remain insufficient, however, and in most cases CKD leads to ESRD.

Stem cell-based therapy is a new strategy for treating chronic kidney injury and may be more effective than single-agent drug therapy because stem cells exhibit highly versatile responses to their environments. Stem cells may secrete cytokines within injured kidneys and participate in endothelial cell proliferation or angiogenesis to facilitate renal regeneration [3, 4]. Increasing evidence has suggested there is therapeutic potential in using mesenchymal stem cells derived from bone marrow to treat chronic kidney injury [5–10]. The important challenge of clinical translation is the risk for long-term maldifferentiation. This leads to the necessity of applying progenitor or stem cells that are derived from kidney to treat chronic kidney injury.

Mouse kidney progenitor-like cells (MKPCs) isolated from *Myh9*-targeted mutant mice have exhibited pluripotent activity, both in vitro and in vivo [11]. As we have demonstrated previously, these cells are renoprotective when injected directly into the kidney of a mouse with acute kidney injury. We determined whether weekly injections of MKPCs slowed the progression of CKD among mice following five-sixths nephrectomy. In this study, we provide evidence that the antifibrotic activity of MKPCs is mediated by a novel endocrine mechanism of action, demonstrating that conditioned medium derived from MKPCs inhibits transforming growth factor beta (TGF-β)-induced endothelial–mesenchymal transition (EndoMT) in vitro. We further demonstrate the relevance of these findings in vivo, showing that intravenous administration of MKPCs could protect five-sixths nephrectomized mice from renal fibrosis via preservation of angiogenic processes.

## Methods

### Isolation of MKPCs

The MKPCs were isolated from the kidneys of 2-month-old *Myh9-*targeted mutant mice and cultured as described previously [11]. Briefly, kidneys were perfused in vivo with saline to flush out the blood from the kidney, dissected, minced, and digested with collagenase 0.3 % and trypsin 0.3 % at 37 °C for 30 minutes in a shaking water bath. After passing through 100 μm mesh to remove undigested chunk, glomeruli, and large renal tubules, the filtered fraction was homogenized in a Dounce homogenizer for 10 strokes and then 40 μm mesh was used to remove smaller renal tubules and cell aggregates. The filtered fraction containing mainly single cells was washed in a medium that consisted of DMEM-LG (Dulbecco's Modified Eagle Medium low glucose), 100 U/ml penicillin, 100 μg/ml streptomycin, and 100 μg/ml gentamicin with 10 % CCS (comic calf serum). Similar cell preparations from C57BL/6J mice were used to determine the level of autofluorescence. The cells prepared from *Myh9*-targeted mutant mouse that express green fluorescence 10-fold higher than those from C57BL/6J mouse were sorted by fluorescence-activated cell sorting (FACS) using FACSAria Cell Sorter (BD Biosciences, New Jersey, USA) equipped to sort green fluorescent protein (GFP). After sorting, GFP-positive cells were plated and cultured on plastic plates in DMEM-LG with 100 U/ml penicillin, 100 μg/ml streptomycin, and 10 % CCS (Hyclone, Pittsburgh, USA) at 37 °C in the presence of 5 % CO_2_.

### Mouse care

Female C57BL/6 mice 8–10 weeks old of approximately similar weights (20–25 g) were housed under pathogen-free conditions. All animal experiments were performed after attaining approval from the Institutional Animal Care and Use Committee of Kaohsiung Veterans General Hospital and according to the Guidelines for the Care and Use of Laboratory Animals of the National Research Council.

### CKD mouse model and MKPC treatment

We developed a mouse CKD model using the following modified protocol. Surgery was performed after anesthetizing the mice with a mixture of ketamine (100 mg/ml) and xylazine (25 mg/ml). A 1:10 dilution of the stock solution in saline was intraperitoneally administered at 0.02 ml/g body weight. Lateral dorsal longitudinal incisions were made to expose the right-side kidney. The upper and lower poles of the right kidney were amputated using electrocoagulation. Left nephrectomy was performed 1 week later. The left kidney was removed and the vascular pedicle was ligated at the hilum using 4–0 silk. The time of left nephrectomy marked the onset of chronic kidney injury. For the MKPC treatment, in vitro expanded MKPCs (2.5 × 10^5^ cells in 150 μl of 0.9 % saline) were intravenously administered to the CKD mice through the tail vein each week from weeks 5 to 10. The control mice underwent an identical five-sixths nephrectomy protocol and intravenously received 0.9 % saline at the same volume used in the cell therapy (Additional file 1: Figure S1).

Blood pressure was measured in anesthetized mice using an automated tail-cuff manometer system (MK-2000ST; Muromachi Kikai, Tokyo, Japan). Blood pressure was measured at least three times and averaged. Blood was sampled via the retro-orbital sinus of the mice. After sacrificing the mice, one hemisection of the kidney was obtained and processed for formalin fixation followed by paraffin embedding or section freezing. The sections were stained with hematoxylin and eosin, periodic acid–Schiff (PAS), Masson’s trichrome, and silver, and then histologically examined for morphological changes. Another hemisection of kidney was also frozen using liquid nitrogen and maintained frozen for further use.

Renal function was assessed by measuring blood urea nitrogen (BUN), serum creatinine, urine protein, and urine creatinine with a quantitative colorimetric assay kit (Sigma, St. Louis, MO, USA). Serum cystatin C was measured with an enzyme-linked immunosorbent assay (ELISA) kit (Mouse Cystatin C DuoSet ELISA; R&D, Minneapolis, MN, USA). The data are expressed as mean ± standard deviation (SD). The groups were compared based on the results of nonparametric testing. *P* <0.05 indicated the presence of a significant difference.

### Immunohistochemistry and immunofluorescence

The fixed kidney sections were deparaffinized in xylene and rehydrated through a graded ethanol series to water. After blocking the samples with 10 % normal horse serum in phosphate-buffered saline (PBS), the slides were stained with primary antibodies overnight at 4 °C, and then biotinylated with secondary antibodies for 30 minutes and diaminobenzidine reagent (Vector Laboratories, Burlingame, CA, USA) for 5 minutes. The primary antibodies used were mouse anti-alpha-smooth muscle actin (anti-αSMA; Dako Cytomation), mouse monoclonal anti-F4/80 (AbD Serotec, Raleigh, NC, USA), mouse monoclonal anti-Ki67 (Upstate, New York, USA), mouse monoclonal anti-GFP (Santa Cruz Biotechnology, Inc.), and anti-CD31 (Abcam, Cambridge, UK ). N-Histofine® Simple Stain™ Mouse MAX PO (Nichireibiosciences, Tokyo, Japan) was used as secondary antibody. In immunofluorescence, mouse anti-human smooth muscle actin (Dako Cytomation, Carpinteria, CA , USA) and rabbit anti-mouse CD31 (Santa Cruz, Texas, USA) were used as primary antibodies. Goat anti-mouse IgG (Molecular Probes) and goat anti-rabbit IgG (Molecular Probes, Oregon, USA) were used as secondary antibodies.

Histological sections (4 μm thick) were stained with Masson’s trichrome, hematoxylin and eosin, or silver, and subsequently examined using light microscopy to determine the levels of glomerular injury, interstitial fibrosis, and tubular atrophy. The extent of glomerulosclerosis was evaluated at 14 and 17 weeks after the five-sixths nephrectomy. A glomerulosclerosis index was derived for each animal by examining at least 20 glomeruli at × 400 magnification. The severity of glomerulosclerosis was expressed on an arbitrary scale from 0 to 2: grade 0 = normal glomeruli; grade 1 = mild/moderate segmental glomerular hyalinosis/sclerosis involving <50 % of the glomerular tuft; and grade 2 = diffuse glomerular hyalinosis/sclerosis involving ≥50 % of the tuft. The resulting index for each animal was expressed as the mean of all scores obtained.

The fractional area of the interstitial fibrosis in the renal cortex was determined using morphometry, involving a video camera connected to an image analyzer (Image-Pro Plus; Olympus,Tokyo, Japan). In each renal cortex, 20 grid fields (each 0.145 mm^2^ in area) were evaluated. The interstitial areas were first manually circled on a video screen and then determined using computerized morphometry. Tubular atrophy was defined based on thick, irregular tubular basement membranes exhibiting a simplified epithelium. The extent of tubular atrophy was expressed based on the total area of atrophic tubules divided by the entire interstitial area. In each animal cortex, 20 grid fields were evaluated. The resulting index for each animal was expressed as the mean of all scores obtained.

For quantification of α-SMA, F4/80 or Ki67 positively stained cells, cells were counted from 10 random cortical fields (×200 magnification) in each section, and the numbers were averaged for each section. To assess the capillary density, the mean area fraction of CD31-positive peritubular capillaries in each visual field at × 200 magnification in the light microscope were counted. Images were analyzed by Image Pro software (Media Cybernetics, Rockville, MD, USA) from 10 random fields in each section, and the numbers averaged for each section.

### Tracking GFP-positive cells

The MKPCs were injected into the tail veins of mice 5 weeks after they underwent five-sixths nephrectomy. The animals were sacrificed 4 hours, 16 hours, 1 day, 2 days, 7 days, or 28 days after the MKPC injection. The kidneys were perfused with saline to flush out blood, dissected, minced, and digested with 0.3 % collagenase and 0.3 % trypsin at 37 °C for 30 minutes in a shaking water bath. After being passed through 40 μm mesh to remove the cell aggregates, the cells were recovered in a medium that consisted of DMEM-LG (Gibco, Life Technologies, NY, USA ), 100 U/ml penicillin (Gibco), 100 μg/ml streptomycin (Gibco), and 100 μg/ml gentamicin (Gibco) with 10 % CCS (Hyclone). The cells subsequently underwent flow cytometry (FACSCalibur; BD Biosciences). Similar cell preparations from C57BL/6J mice were used to determine the level of autofluorescence, and cells from Myh9-targeted mutant mice were used as positive controls.

We quantified GFP-containing cells in the lungs after intravenous injection of 2.5 × 10^5^ MKPCs in week 5 into mice subjected to five-sixths nephrectomy, and the lungs were harvested, lysed with collagenase, and centrifuged to obtain cell suspension. Quantification of GFP-containing cells was made by flow cytometry. Lung obtained from a Myh9-targeted mutant mouse was used as a positive control while lung from a C57BL/6 mouse was used as a negative control.

### Preparing the conditioned medium

The MKPCs were plated at 5000 cells/cm^2^ and incubated in the aforementioned culture medium for 1 day. The attached cells were washed three times with PBS, and the medium was replaced with DMEM to generate a serum-free and glucose-free medium. The cells were then incubated for 2 days and the conditioned medium was harvested.

### Endothelial cell culture

Mouse pancreatic microvascular endothelial cells (MMECs) were grown in a 5 % CO_2_ atmosphere at 37 °C in DMEM containing 10 % fetal bovine serum. Recombinant human TGF-β (R&D Systems) with or without conditioned medium was added to the cell cultures for 14 days at concentrations of 1 and 5 ng/ml. The treated cells subsequently underwent western blotting.

### Culture under hypoxic conditions

The MMECs were expanded under standard normoxic conditions for cell cultures at 37 °C in a humidified incubator containing 21 % O_2_, 5 % CO_2_, and 74 % N_2_. To examine hypoxia-induced MMEC cell death, the cells were plated in a 6 cm dish and incubated for 2 days under standard conditions. Incubation was continued under normoxic or hypoxic conditions with or without conditioned medium for 24 and 28 hours. With regards to the hypoxic conditions, the cultures were placed in an atmosphere-controlled CO_2_ incubator containing a gas mixture composed of 94 % N_2_, 5 % CO_2_, and 1 % O_2_. The nitrogen was fed from a tank of liquid N_2_. Following the ischemic incubation, the cell cultures were removed from the hypoxic chamber, rinsed with PBS, observed under a microscope, and collected for immunocytochemistry or flow cytometry. The experiments were repeated in triplicate.

### Immunocytochemistry

The cells were fixed with 4 % paraformaldehyde solution and quenched with 50 mmol/l ammonium chloride. After permeabilization with 0.1 % (v/v) Triton X-100 and blocking with 1 % horse serum, the cells were incubated with primary antibodies for 1 hour, and then with fluorophore-conjugated secondary antibodies for 40–60 minutes at room temperature. The employed antibodies were anti-CD31 and anti-αSMA. Secondary antibodies were also employed and 4′,6-diamidino-2-phenylindole (DAPI) staining was used to facilitate the detection of the nuclei.

### Flow cytometry

For flow cytometry, the collected cells were suspended in PBS. A minimum of 150,000 cells were collected for all analyses. Gating was constructed based on the negative controls, and compensation controls were included in all analyses. Population percentages and numbers were generated for the gated populations in each experiment using WinMDI software version 2.8 (The Scripps Research Institute, La Jolla, CA, USA). For the apoptosis assay, we employed an annexin V binding assay. Briefly, cells in 6 cm culture plates were collected and double-stained with annexin V–fluorescein isothiocyanate (FITC) and propidium iodide (PI) using an annexin V–FITC Apoptosis Detection Kit (Roche, New York, USA). The stained cells were processed by conducting flow cytometry analysis using a FACSCalibur cytometer (BD Biosciences). The fraction of cells that was annexin V-positive and PI-positive was considered apoptotic, and the fraction of cells that was annexin V-negative and PI-positive was considered necrotic. The sum of both fractions was considered the cell death ratio.

### Western blot analysis

Cell culture samples were sonicated and resuspended in 0.4 ml RIPA lysis buffer. The protein concentrations were estimated using a detergent-compatible protein assay kit (Bio-Rad, Hercules, CA, USA). Samples containing 18 μg total protein were blotted with anti-CD31 and anti-αSMA. The blots were then incubated with peroxidase-conjugated goat anti-rabbit immunoglobulin G (IgG) or goat anti-mouse IgG (Santa Cruz), and the bound antibody was detected using ECL (Visual Protein Biotechnology Taipei, Taiwan) and captured using the Epson Perfusion V350 Photo system (Epson, Long Beach, CA, USA). Densitometry analysis was performed using a UN-SCAN-IT gel 6.1 analyzer (Silk Scientific Corporation, Orem, UA, USA). To control the loading, the blots were incubated with actin antibody (Santa Cruz).

## Results

### MKPCs ameliorate renal dysfunction in the five-sixths nephrectomy model

**Fig. 1:**
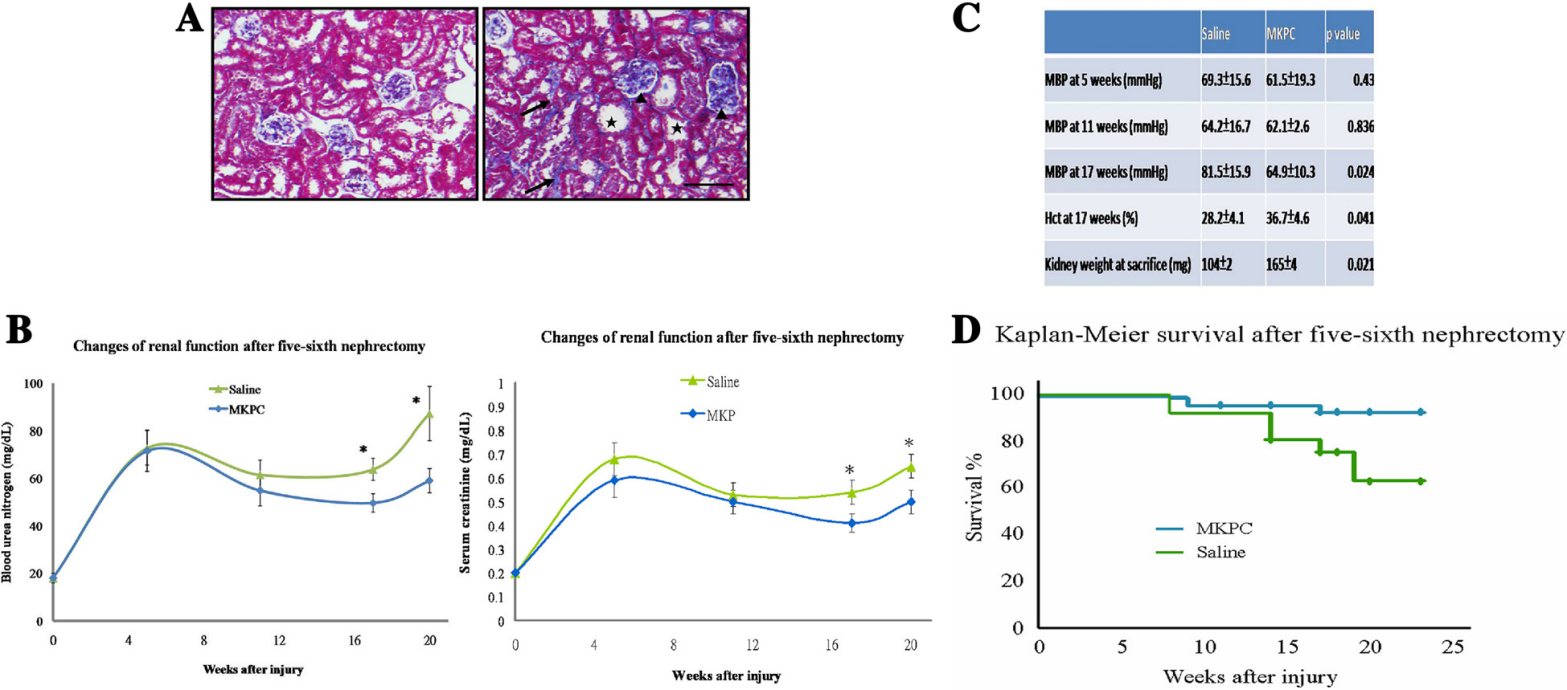
MKPC injections improve renal function and prolong survival. **a** Wild-type mice present normal renal morphology without any abnormalities (*left*) compared with mice receiving five-sixths nephrectomy that exhibit chronic kidney injury (*right*), as shown in representative Masson’s trichrome staining pictures of kidney sections from mice 17 weeks after five-sixths nephrectomy. *Black arrows* point to interstitial fibrosis, *stars* indicate tubular atrophy, and *arrowheads* represent glomerular sclerosis. Scale bar: 100 μm. **b** Serial BUN and serum creatinine levels measured in five-sixths nephrectomized mice (*n* = 6 in each group) that received saline (*triangles*) or MKPC (*diamonds*). MKPC administration weekly from weeks 5 to 10 after establishment of chronic kidney injury significantly improves renal function at 17 and 21 weeks, whereas saline-treated mice show no such effect. *P <0.02, MKPC-treated vs. saline-treated mice. **c** Five-sixths nephrectomized mice treated with MKPCs feature lower blood pressures, higher hematocrit, and larger kidney size at 17 weeks compared with mice treated with saline (*n* = 6 in each group). **d** MKPC treatment prolongs survival in mice with chronic kidney injury. Kaplan–Meier survival in the MKPC group vs. the saline group. The survival differences between these two groups were tested with the log-rank test that shows a statistical significant difference (*P* = 0.01). Mice given MKPCs survived significantly longer than PBS-treated mice. **b**, **c** Data presented as mean ± SD. *Hct* hematocrit, *MBP* mean blood pressure, *MKPC* mouse kidney progenitor-like cells

MKPCs were isolated and characterized before being administered to the animals by the methods used in our previous study [11]. To determine whether MKPCs influenced renal function, we measured the BUN levels in mice that underwent the five-sixths nephrectomy to induce CKD and subsequently received injections of either MKPCs or saline. The mice developed CKD, exhibiting increased interstitial fibrosis, tubular atrophy, and glomerular sclerosis (Fig. 1a). The remnant kidneys in the mice receiving saline yielded significantly increased serum BUN and creatinine levels from weeks 11 through 21. The injection of MKPCs protected renal function, and was demonstrated by the significantly lower BUN and creatinine values at weeks 17 and 21 (Fig. 1b) in the MKPC-treated mice compared with the BUN and creatinine values in the saline-treated mice. To exclude the possibility that saline alone already has an ameliorating effect on renal function, we have measured renal function in mice that underwent five-sixths nephrectomy without receiving any injection. As compared with mice receiving weekly saline intravenous injection from weeks 5 to 10, mice without any injection exhibited similar renal function (Additional file 2: Figure S2). Moreover, mice of the MKPC group received MKPCs dissolved in saline, the same amount as those in mice of the saline group. This eliminates the confounding effect of saline injection in our study. Blood pressure in the normal mice was 65.3 ± 14.7 mmHg. The mice treated with MKPCs displayed not only decreased mean blood pressure levels, but also increased hematocrit levels and larger kidney sizes (Fig. 1c).

### MKPCs prolong the survival of mice with chronic kidney injuries

We further tested whether the MKPC treatment affected survival, which is the critical outcome following chronic kidney injury. Figure 1d shows the survival curves of the mice with chronic kidney injuries treated with saline and those of the mice treated with MKPCs (*n* = 50). The mice injected with the MKPCs survived significantly longer than the saline-treated mice (*P* = 0.01). At week 11, only two of the mice (4 %) who were injected with MKPCs had died, whereas 14 % of the mice in the saline group had died. At week 21, the percentage of surviving MKPC-treated mice remained at 94 %, whereas only 74 % of the saline-treated mice were still alive. These results indicate that the MKPCs both rescue renal injuries and prolong survival in mice after five-sixths nephrectomy.

### MKPCs preserve renal morphology in the five-sixths nephrectomy model

**Fig. 2:**
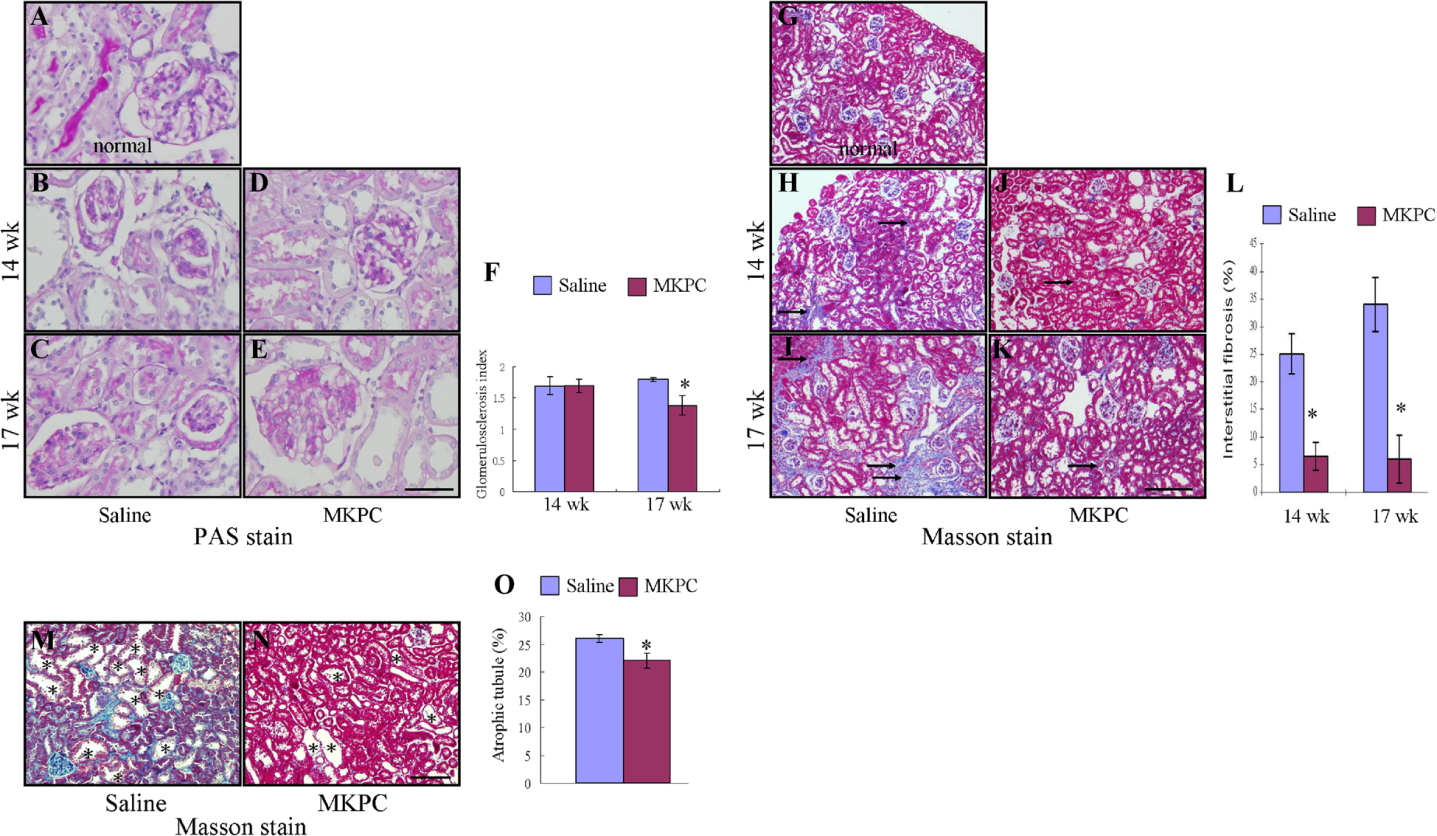
MKPC injections significantly ameliorate the histologic features of chronic kidney injury in five-sixths nephrectomized mice. **a**–**f** Representative photomicrographs of PAS-stained kidney section before establishment **a**, and at 14 weeks **b, d** and 17 weeks **c, e** of CKD in mice treated with saline (*left*) or MKPCs (*right*). The glomeruloslcerosis was more prominent in the saline group than in the MKPC group 17 weeks after five-sixths nephrectomy and was expressed by the glomerulosclerosis index **f**. Scale bar: 50 μm. **g**–**l** Representative photomicrographs of Masson’s trichrome-stained kidney section before establishment **g**, and at 14 weeks **h**, **j** and 17 weeks **i**, **k** of CKD in mice treated with saline (*left*) or MKPCs (*right*). Interstitial fibrosis (*arrow*) was more extensive in the saline group **h, i** than in the MKPC group **j**, **k**. **l** Fractional interstitial fibrosis area. Scale bar: 200 μm. **m**–**o** Representative photomicrographs of Masson’s trichrome-stained kidney section at 17 weeks of CKD in mice treated with saline **m** or MKPCs **n**. *Five-sixths nephrectomized mice receiving MKPC showed less atrophic tubules, which was expressed by the fractional area of atrophic tubules **o**. Scale bar: 100 μm. *P <0.05 MKPC-treated vs. saline-treated group. Data presented as mean ± SD. *Masson* Masson’s trichrome stain, *MKPC* mouse kidney progenitor-like cells, *PAS* periodic acid-Schiff stain

Improved renal function following the MKPC treatment was also associated with enhanced preservation of the renal structure (Fig. 2). The kidneys treated with only saline had increased levels of glomerular sclerosis (Fig. 2b, c), extensive interstitial fibrosis (Fig. 2h, i), and marked tubular atrophy (Fig. 2m). By contrast, the kidneys treated with MKPCs exhibited markedly reduced histological features of renal fibrosis. The glomerular sclerosis index was also significantly lower among the MKPC group (Fig. 2f). In addition to the lesions observed in the glomeruli, the fractional interstitial area (Fig. 2l) and atrophic tubule area (Fig. 2o) also decreased in mice injected with the MKPCs by week 17.

### In vivo tracking of MKPCs

**Fig. 3:**
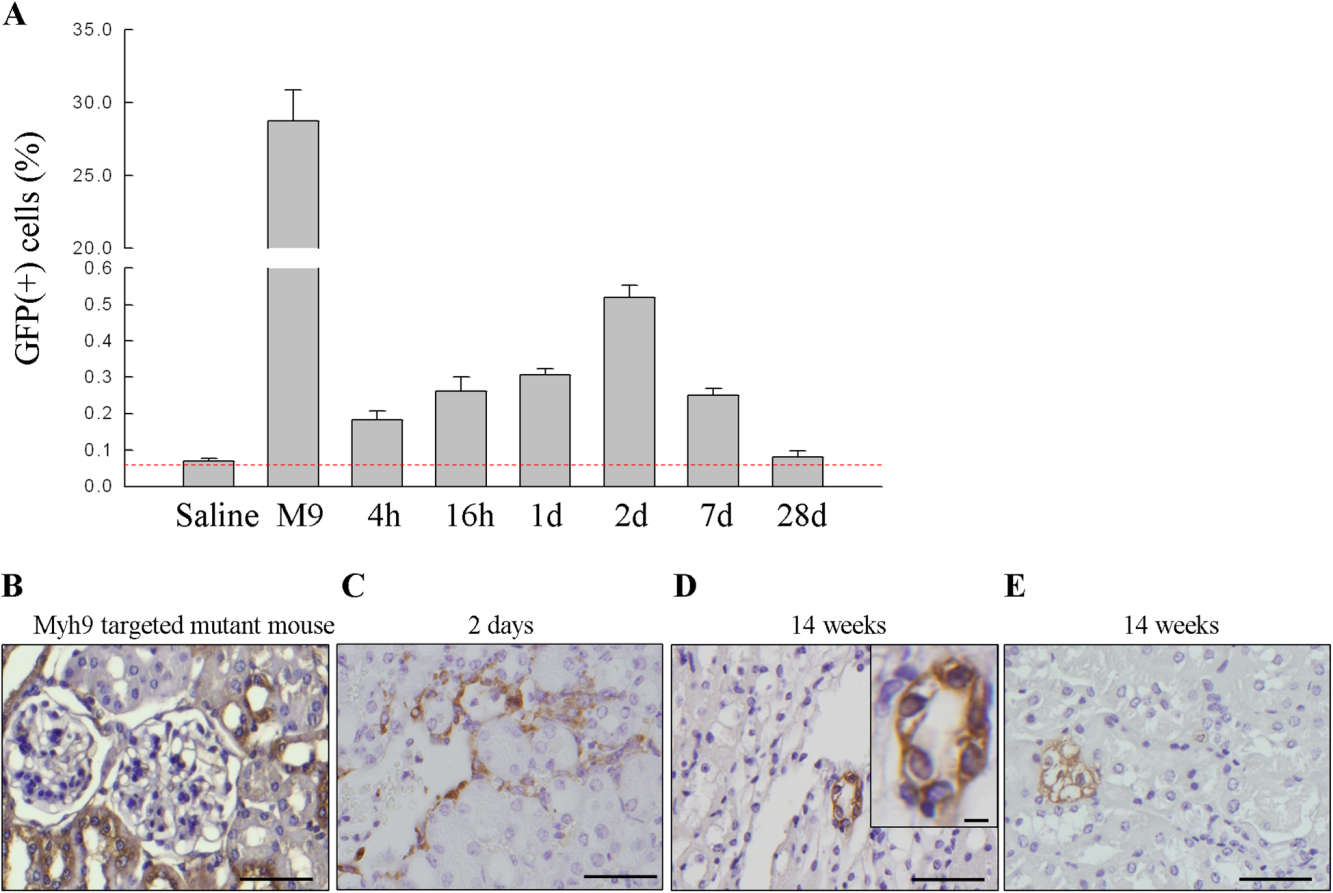
Presence of GFP-positive cells in the kidney of five-sixths nephrectomized mice. **a** As detected by FACS, the presence of GFP-positive cells peaked at 2 days and declined at 7 days after injection. After 28 days, few GFP-positive cells were detected in the kidney. *Myh9*-targeted mutant mice were used as a positive control and five-sixths nephrectomized mice treated with saline were used as a negative control. Data presented as mean ± SD. **b**–**e** Representative photomicrographs of GFP-stained kidney section 2 days and 14 weeks of CKD in mice treated with MKPCs. Kidney from Myh9-targeted mutant mouse was used as a positive control **b**. GFP-positive cells were found in some areas of the interstitium at the second day **c**, formed a tubule-like structure in the medulla **d** (inlet with higher magnification), or remained in the interstitium at 14 weeks. Scale bar: 50 μm. *d* days, *GFP* green fluorescent protein, *h* hours, *M9* *Myh9*-targeted mutant mice, *Saline* five-sixths nephrectomized mice treated with saline

To quantify the GFP-positive cells present in the kidneys after the five-sixths nephrectomy, the mice were injected once with 2.5 × 10^5^ MKPCs 5 weeks after establishing chronic kidney injury. The cells containing GFP were quantified using flow cytometry and the results are presented in Fig. 3a. The presence of GFP-positive cells in the remnant kidneys was transient, increasing at 4 hours, peaking at 2 days, and declining at 7 days. By 28 days after a single injection of MKPCs, scant GFP-positive cells were detected in the remnant kidneys. To evaluate whether the MKPCs could be incorporated into parts of the remnant kidneys, we traced the MKPCs by staining the GFP in mice 14 and 17 weeks after the five-sixths nephrectomy. Only a limited area of the medulla exhibited evidence that the injected MKPCs were being incorporated into renal tubules (Fig. 3d) or were scattered in the interstitium (Fig. 3e). It is widely accepted that injection of cells via the tail vein leads to accumulation of cells in the lungs. To eliminate the long-term effect of MKPCs in the lungs, we quantified GFP-positive cells in the lungs after intravenous injection of 2.5 × 10^5^ MKPCs in week 5 into mice subjected to five-sixths nephrectomy. Quantification of GFP-positive cells was made by flow cytometry and the result is shown in Additional file 3: Figure S3A. The increase in the number of GFP-positive cells in the lung peaks at 4 hours, declines at 16 hours, reaches a plateau at 1 day, and is negligible until 9 weeks after MKPC injection (i.e., the 14th week after five-sixths nephrectomy). These results were supported by immunohistochemistry of the lung which shows GFP-positive cells in the alveoli at the first day (Additional file 3: Figure S3B). No GFP-positive cells were found 14 weeks after nephrectomy. These results suggest that the presence of MKPCs in the lung might be transient.

These results indicate that the effect of MKPCs on the preservation of renal function after five-sixths nephrectomy was not mediated through the direct engagement of the stem cells. We further tested the effect of conditioned medium on renal progression. Five weeks after five-sixths nephrectomy, mice received treatment with weekly intravenous injection of conditioned medium, MKPCs or saline for 6 weeks. At 17 and 21 weeks, treatment with conditioned medium or MKPCs resulted in similar but significantly better renal function compared with treatment with saline (both *P* <0.05; Additional file 4: Table S1).

### MKPCs reduce the infiltration of inflammatory cells and promote the proliferation of tubule cells in the five-sixths nephrectomy model

**Fig. 4:**
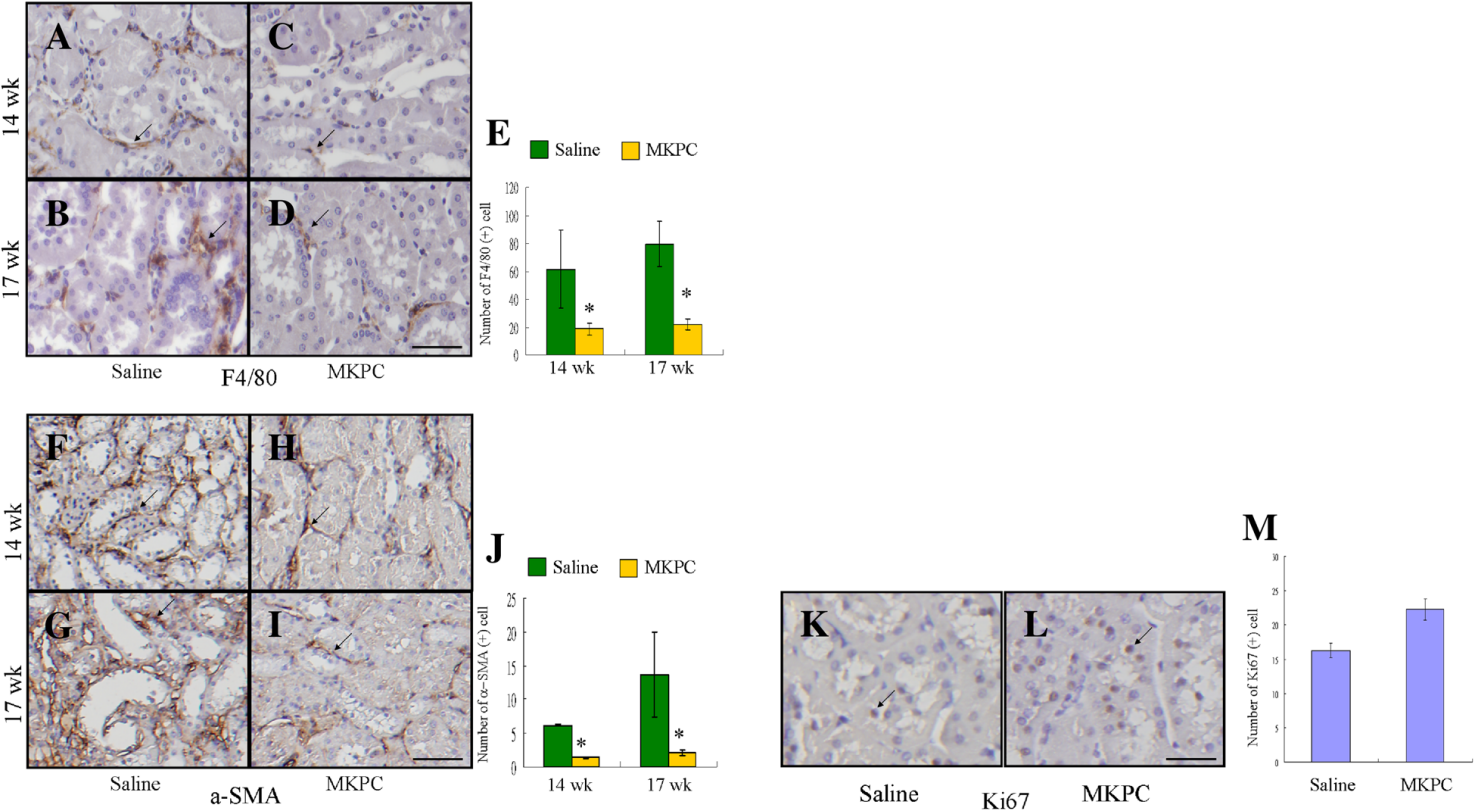
MKPC injection decreases the recruitment of inflammatory cells and promotes proliferating tubule cells in five-sixths nephrectomized mice. **a**–**j** Representative photomicrographs illustrate F4/80 and α-SMA expression in the renal tissue of mice receiving saline or MKPC 14 and 17 weeks after five-sixths nephrectomy. Lower numbers of F4/80-positive cells **a**–**e** (*arrow*) and α-SMA-positive cells **f**–**j** (*arrow*) accumulate in the interstitium of mice treated with MKPCs compared with those injected with saline. **k**–**m** Ki67-positive cells (*arrow*) are more frequently found in the cortical tubules in mice receiving MKPC injection 14 weeks after chronic kidney injury. Scale bar: 50 μm. *P <0.05, MKPC-treated vs. saline-treated group. Data presented as mean ± SD. *α-SMA* alpha-smooth muscle actin, *MKPC* mouse kidney progenitor-like cells, *Saline* five-sixths nephrectomized mice treated with saline, *wk* weeks

We evaluated the presence of two major cell types in areas of renal fibrosis. As shown in Fig. 4, the number of macrophages expressing F4/80 in the tubulointerstitium was significantly lower in the mice treated with MKPCs than that in the saline-treated mice. The decreased infiltration of macrophages in the kidneys treated with MKPCs was associated with a decreased number of myofibroblasts in the interstitium. In addition, the mice treated with MKPCs displayed significantly higher proliferation of tubular cells 14 weeks after the five-sixths nephrectomy than the saline-treated mice (Fig. 4k, l).

### Effects of MKPCs on vascular phenotypes

**Fig. 5:**
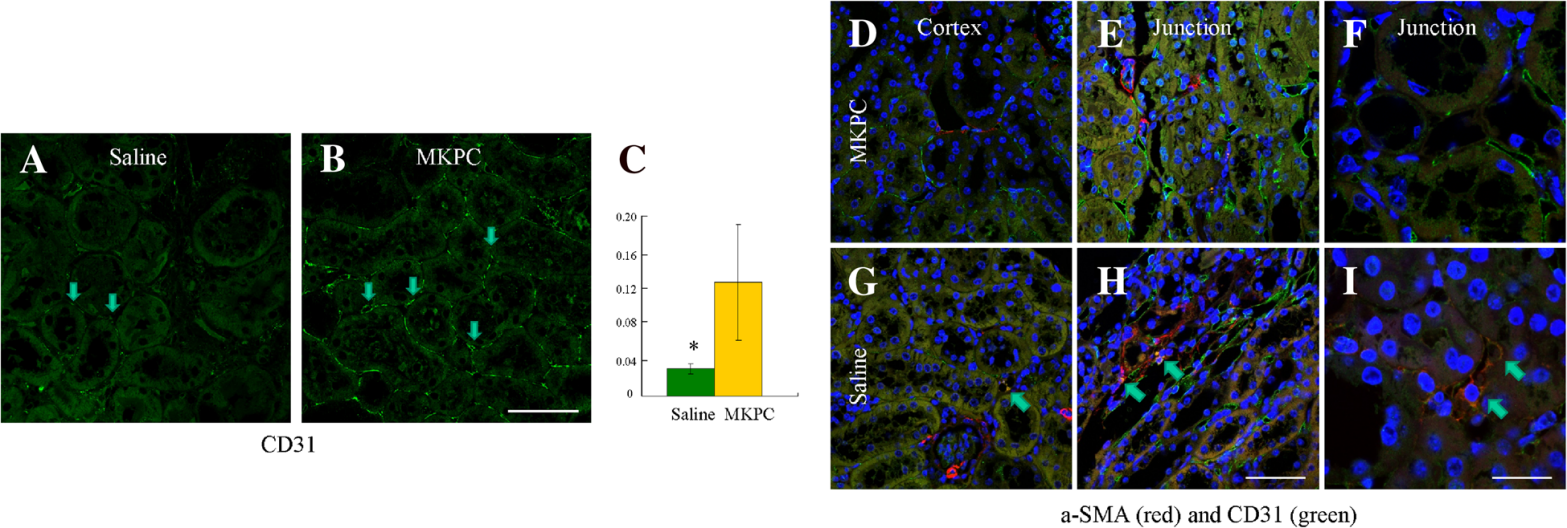
MKPC injection preserves capillary density and prevents EndoMT in five-sixths nephrectomized mice. **a**–**c** Capillary rarefaction occurs in five-sixths nephrectomized mice treated with saline **a**, but MKPC injection preserves capillary density **b** at 17 weeks after establishment of chronic kidney injury. Scale bar: 50 μm. **d**–**i** Confocal microscopy demonstrates α-SMA (*red*) and CD31 (*green*) staining in 17-week nephrectomized mice. *Arrows* indicate α-SMA and CD31 double-positive cells in the capillaries. EndoMT occurs in the interstitium of cortex and cortico-medullary junction from five-sixths nephrectomized mice treated with saline. The MKPC injection prevents EndoMT. Scale bar: 50 μm **h**, 20 μm **i**. *α-SMA* alpha-smooth muscle actin, *Junction* cortico-medullary junction of the kidney, *MKPC* mouse kidney progenitor-like cells, *Saline* five-sixths nephrectomized mice treated with saline

We examined how MKPCs affected the microvasculature of kidneys after the five-sixths nephrectomy. As shown in Fig. 5a, b, the capillary rarefaction was significantly reduced in the remnant kidney treated with the MKPCs. EndoMT is considered to be an origin of renal fibroblasts in CKD. Figure 5d–i show sections of various regions of kidneys costained for α-SMA and CD31. Substantial amounts of α-SMA-positive and CD31-positive cells were observed in the corticomedullary junction of the kidney treated with saline. By contrast, no α-SMA-positive and CD31-positive cells were observed in the MKPC-treated mice. This suggests that MKPCs prevented EndoMT following five-sixths nephrectomy.

### Effects of MKPC-conditioned medium on angiogenic processes in vitro

**Fig. 6:**
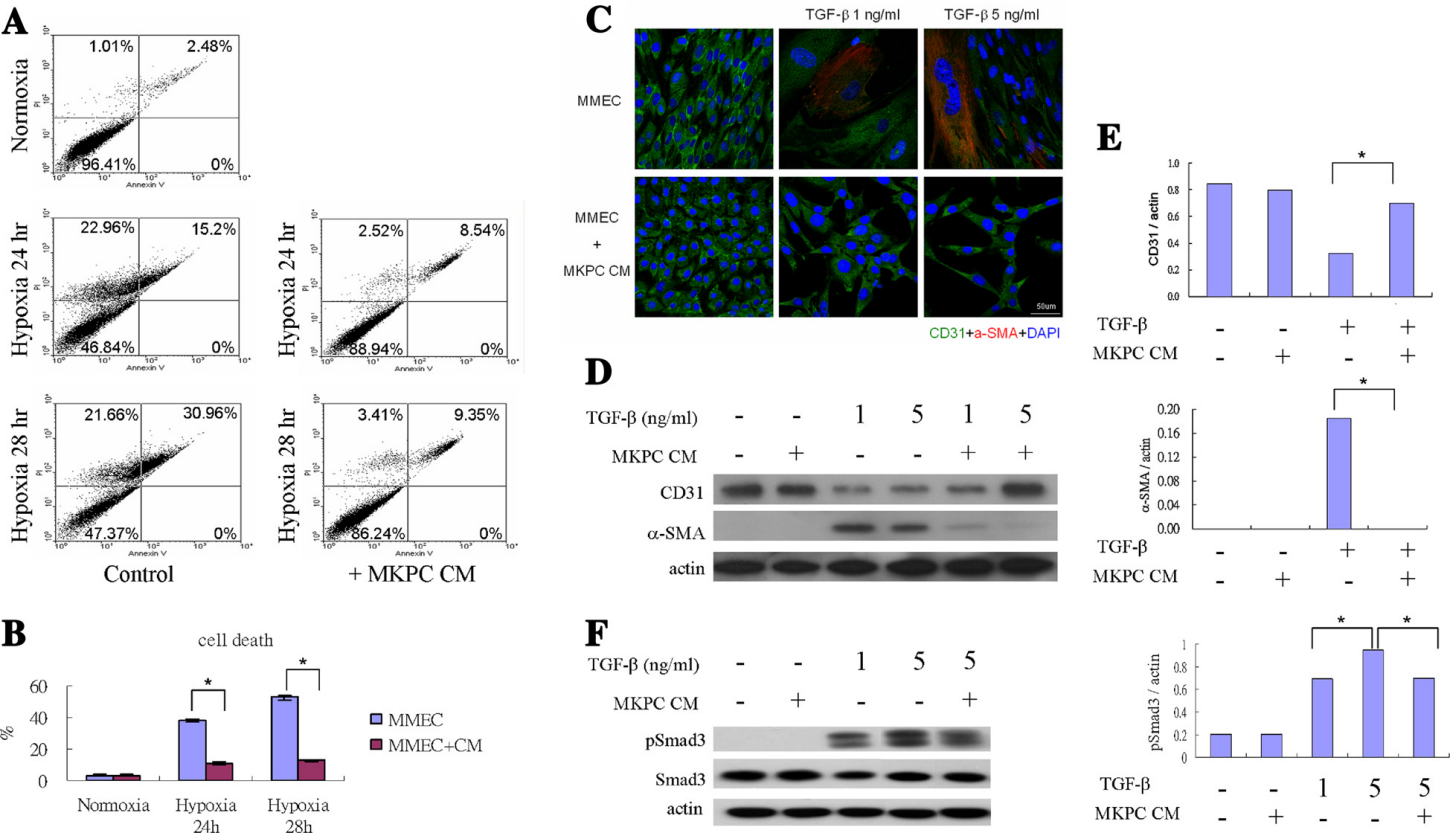
MKPC conditioned medium promote angiogenic process in vitro. **a**, **b** Conditioned medium from MKPCs ameliorates endothelial cell death following hypoxic culture condition. Endothelial cells (MMECs) were treated with or without conditioned medium from MKPCs (*MKPC CM*) under normoxic or hypoxic culture conditions for 24 or 28 hours. Cell death was measured by Annexin V and PI staining detected by flow cytometry. Each experiment was repeated three times. Cells in the *upper left* and *upper right* plots were considered death cells. **c**–**f** Conditioned medium from MKPCs prevents TGF-β-induced EndoMT in vitro. Confocal microscopy demonstrates DAPI (*blue*), CD31 (*green*), and α-SMA (*red*) in MMECs cultured with or without MKPC CM and with or without TGF-β for 14 days **c**. Representative western blot and relative bar graph analysis of CD31, α-SMA **d**, **e**, Smad3 and pSmad3 **f** protein level in MMECs after various treatments. Fibroblast-like change of MMECs was observed following TGF-β treatment, but the change was reduced in MMECs incubated with MKPC CM **c**. TGF-β treatment (5 ng/ml) caused increased α-SMA protein expression and decreased CD31 protein expression **d**, **e**. MKPC CM inhibited phosphorylation of Smad3 in MMEC after 1 hour of TGF-β stimulation **f** and attenuated these TGF-β-induced EndoMT responses. *P <0.05 compared with control or between two groups. *α-SMA* alpha-smooth muscle actin, *DAPI* 4′,6-diamidino-2-phenylindole, *MMEC* mouse pancreatic microvascular endothelial cell, *TGF-β* transforming growth factor beta

To determine whether the conditioned medium of MKPCs protected against hypoxia-induced cell death in endothelial cells in vitro, endothelial cells (i.e., MMECs) were cocultured with the conditioned MKPC medium following hypoxia for 24 and 28 hours. As shown in Fig. 6a, the hypoxia dose-dependently enhanced the cell death of endothelial cells (52.6 ± 1.9 % in hypoxia for 28 hours vs. 38.2 ± 0.9 % in hypoxia for 24 hours vs. 3.5 ± 0.3 % in the control group; *P* <0.05; Fig. 6b). Moreover, the conditioned medium from the MKPCs significantly reduced the amount of hypoxia-induced cell death (*P* <0.05 vs. without conditioned medium) after 24 and 28 hours under hypoxic conditions.

TGF-β plays a pivotal role in the development and progression of renal fibrosis, and EndoMT contributes to renal fibrosis. To investigate whether the conditioned MKPC medium could prevent TGF-β-induced EndoMT in vitro, we cultured MMECs in the presence of TGF-β. Fibroblastoid change of cultured MMECs was observed following TGF-β stimulation, whereas the change was milder in MMECs incubated with MKPC conditioned medium (Fig. 6c). Confocal microscopy demonstrated the TGF-β-induced de novo expression of α-SMA (Fig. 6c), a putative marker of myofibroblasts. Concurrently, the MMEC expression of the endothelial cell markers CD31 was lost (Fig. 6d). Confocal microscopy (Fig. 6c) and western blot (Fig. 6d, e) demonstrated that the conditioned medium from the MKPCs could significantly reduce the expression of α-SMA and preserve the expression of CD31 in endothelial cells treated with TGF-β. Moreover, western blot analysis shows that MKPC conditioned medium inhibited TGF-β-induced phosphorylation of Smad3 in MMECs (Fig. 6f). This indicates that the conditioned MKPC medium could prevent TGF-β-induced EndoMT through downregulation of phosphorylated Smad 3 in vitro.

## Discussion

Regardless of its etiology, the process of CKD is generally irreversible and inevitably leads to end-stage renal failure, a condition requiring life-long dialysis or renal transplantation. In this study, we examined how kidney progenitor-like cells positively influenced renal outcome after chronic kidney injury. Although mice died of uremia after experiencing chronic renal injuries, treating these mice with kidney progenitor-like cells saved lives by improving renal fibrosis. Intravenously injecting MKPCs protected the mice from renal function deterioration and subsequent death, by decreasing the EndoMT and preserving angiogenesis following a five-sixths nephrectomy.

Various studies have indicated that stem cells derived from the kidney accelerate the recovery of acute kidney injuries [11–13]. The role of stem cells in the progression of CKD has primarily been observed in studies that applied mesenchymal stem cells in various models such as glomerular basal membrane-associated type IV collagen gene mutations, anti-Thy 1,1-induced glomerulonephritis, unilateral ureteral obstruction, and five-sixths nephrectomy [7, 8, 14–17]. We selected a five-sixths nephrectomy model because it is considered to be a classic model of progressive renal disease characterized by renal inflammation and the gradual development of glomerulosclerosis and tubulointerstitial fibrosis, thereby mimicking CKD in humans [18]. In the study, the MKPC infusion significantly improved renal function and prolonged survival. Anemia and hypertension were also corrected among the MKPC-treated animals, reflecting their enhanced kidney function. To date, no studies have evaluated the potential benefits of treatment with adult kidney progenitor-like cells in CKD models. This result is compatible with previous studies showing the beneficial effect of progenitor cells derived from human fetal kidney on renal function halting disease progression in the five-sixths nephrectomy model [19, 20].

The mechanism by which the injected MKPCs ameliorated renal fibrosis is intriguing and may have resulted from various factors. After the five-sixths nephrectomy and the intravenous injection of renal progenitor-like cells, the engraftment of MKPCs into tubular or endothelial cells was observed to be localized in limited areas rather than widely diffused. Thus, the direct contribution of MKPCs to renal repair could be minor, as indicated by the results, where the MKPCs that were detected 2 days after the injection diminished over time, representing only 0.08 % of the kidney cells at 28 days after a single injection.

Alternatively, experimental studies have indicated that vascular rarefaction in the remnant kidney which occurs as a result of endothelial damage following chronic kidney injury is a critical step toward secondary tissue hypoxia and kidney progression [21–23]. The fibrotic tubulointerstitium, which is a hypoxic environment, exacerbates problems and progressively replaces damaged parenchymal tissue with nonfunctioning scar tissue, leading to prominent capillary rarefaction [24]. In this study, the injected MKPCs protected against endothelial injury in vitro and in vivo. This might facilitate the supply of additional oxygen or regenerative materials to ensure vascular stability during kidney injury, thereby limiting renal fibrosis. A recent study shows that the vasculogenic activity of endogenous mesenchymal stem cells isolated from mice of chronic kidney injury was blunted by uremic toxins [25]. A supply of progenitor-like cells derived from healthy mice kidney might thus correct the defect and promote the angiogenic process in mice with chronic kidney injuries.

Recent evidence has suggested that stem cells downregulate the adhesion of flowing neutrophils or lymphocytes and their subsequent transendothelial migration [26]. We observed evidence that the systemic injection of MKPCs may provide protection by modulating the recruitment of macrophages. Another study indicated that the beneficial effects of MKPCs might be mediated through their ability to supply large amounts of immunomodulatory factors, thereby lessening tissue inflammation in kidney fibrosis [17]. Further studies are required to uncover the relevant mechanisms. Cell therapy with renal stem cells may be a strategy for treating chronic kidney injury. Because of the strong therapeutic potential of stem cell therapies, the mechanisms and long-term safety of this form of therapy must be defined.

The accumulation of activated fibroblasts in affected tissues and the persistence of their elevated biosynthetic functions are crucial determinants of the rate of progression in renal fibrosis. Studies have suggested that the activated fibroblasts in fibrotic kidneys originate from several sources, including the expansion of resident tissue fibroblasts, migration, and the tissue accumulation of bone-marrow-derived fibrocytes from epithelial cells or pericytes that have undergone mesenchymal transition [27, 28]. Endothelial cells can also undergo EndoMT and might be a crucial source of activated fibroblasts participating in renal fibrosis [29, 30]. Our in vitro experiments demonstrated that the endothelial cells activated by TGF-β expressed α-SMA, which is a critical marker of EndoMT. Adding conditioned medium from the MKPCs to the culture system decreased the expression of phosphorylated Smad3 and α-SMA. The results of a recent study have suggested that progenitor-like cells secrete antifibrotic agents, thereby ameliorating kidney and cardiac fibrosis [31]. We speculate that MKPCs can interfere with TGF-β signaling among endothelial cells, thereby protecting these cells from damage and delaying the progression of renal fibrosis.

## Conclusion

In this study, MKPCs ameliorated the progression of fibrosis in a remnant kidney model, prolonging the survival of mice after a five-sixths nephrectomy. We speculate that the modulation of angiogenic responses may be involved in this process. This study elucidates the use of cell therapy in treating chronic kidney injury.

## Additional files

Additional file 1: Figure S1. Showing the time scheme of the experiment. (TIFF 95 kb) Additional file 2: Figure S2. Showing serial BUN levels in five-sixths nephrectomized mice (*n* = 4 in each group) that received saline (*triangles*) or without any injection (*diamonds*). (TIFF 99 kb) Additional file 3: Figure S3. Showing **A** quantification of GFP-positive cells in the lung after intravenous injection of MKPCs in five-sixths nephrectomized mice (*y* axis shows the number of cells, while the *x* axis (FL1-H) shows the fluorescence intensity; M1 is the area of GFP-positive cells) and **B** immunohistochemistry of the lung after intravenous injection of MKPCs into a mouse that underwent five-sixths nephrectomy. Few GFP positive cells were found in the lung at the first day but there were no GFP-positive cells at week 14. (TIFF 2253 kb) Additional file 4: Table S1. Showing that injections of conditioned medium derived from MKPCs improve renal function. (TIFF 144 kb)

## Abbreviations

α-SMA: Alpha-smooth muscle actin
BUN: Blood urea nitrogen
CKD: Chronic kidney disease
DAPI: 4′,6-Diamidino-2-phenylindole
ELISA: Enzyme-linked immunosorbent assay
EndoMT: Endothelial–mesenchymal transition
ESRD: End-stage renal disease
FACS: Fluorescence-activated cell sorting
FITC: Fluorescein isothiocyanate
GFP: Green fluorescent protein
IgG: Immunoglobulin G
MKPC: Mouse kidney progenitor-like cell
MMEC: Mouse pancreatic microvascular endothelial cell
PAS: Periodic acid-Schiff
PBS: Phosphate-buffered saline
PI: Propidium iodide
SD: Standard deviation
TGF-β: Transforming growth factor beta
