# Supplementary figures and images for: Progenitor-like cells derived from mouse kidney protect against renal fibrosis in a remnant kidney model via decreased endothelial mesenchymal transition
Source: Stem Cell Res Ther. 2015 Dec 2;6:239. doi: 10.1186/s13287-015-0241-8 (PMC4668678; doi:10.1186/s13287-015-0241-8)

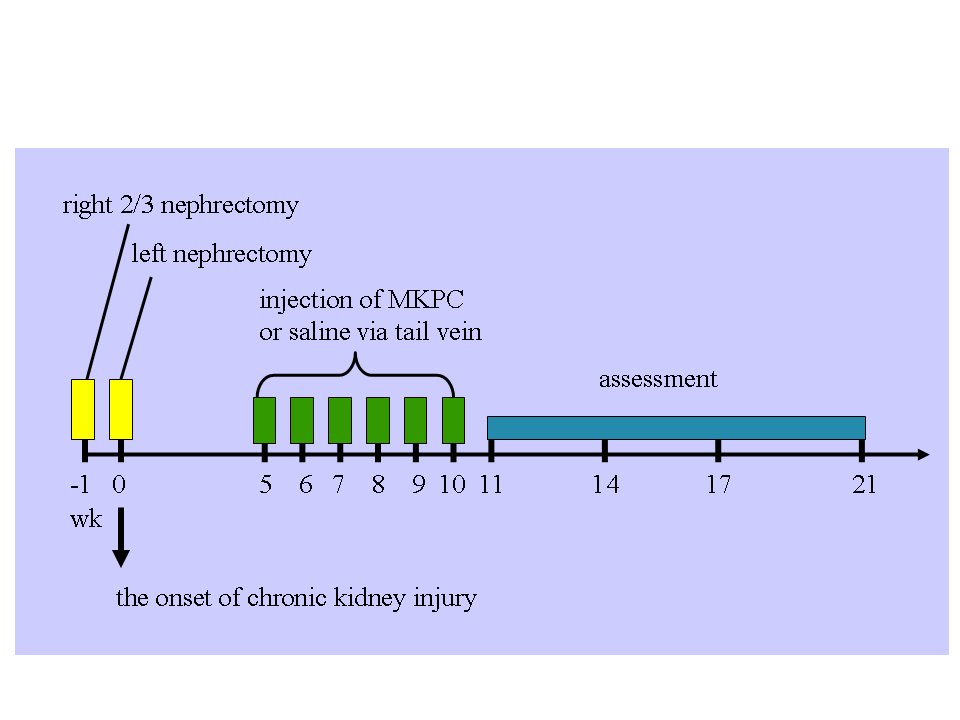

Supplement: Additional file 1: Figure S1. — Showing the time scheme of the experiment. (TIFF 95 kb) [file 13287_2015_241_MOESM1_ESM.tiff]

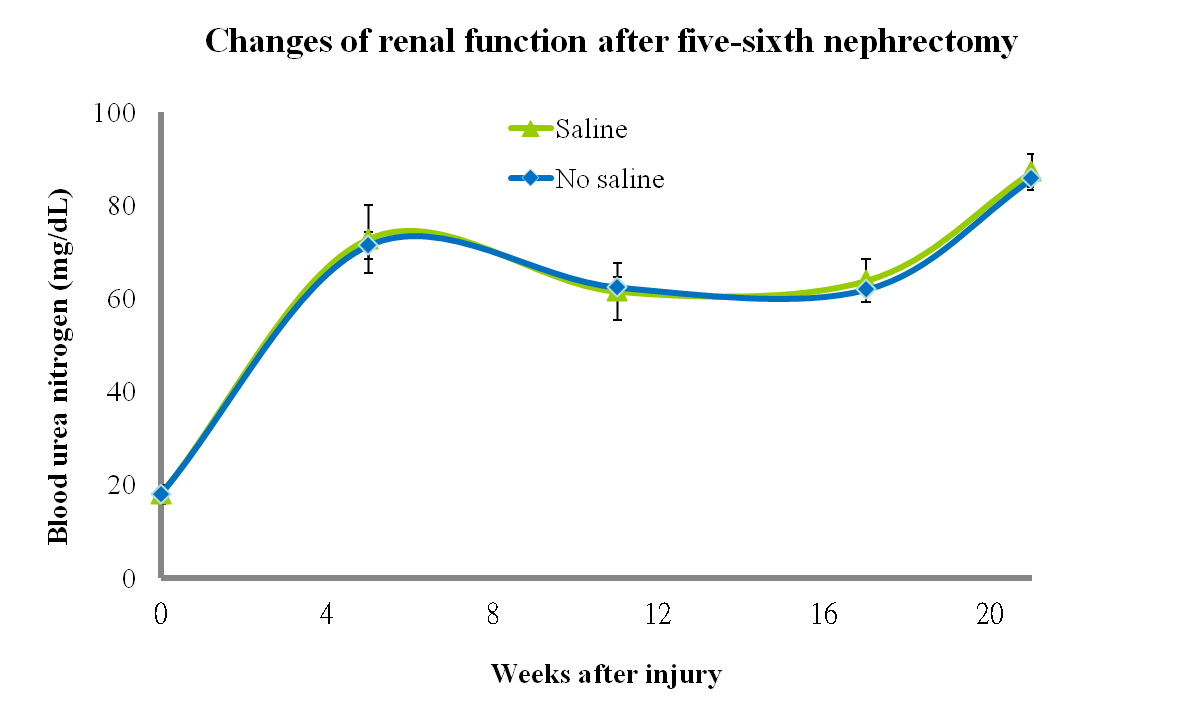

Supplement: Additional file 2: Figure S2. — Showing serial BUN levels in five-sixths nephrectomized mice (n = 4 in each group) that received saline (triangles) or without any injection (diamonds). (TIFF 99 kb) [file 13287_2015_241_MOESM2_ESM.tiff]

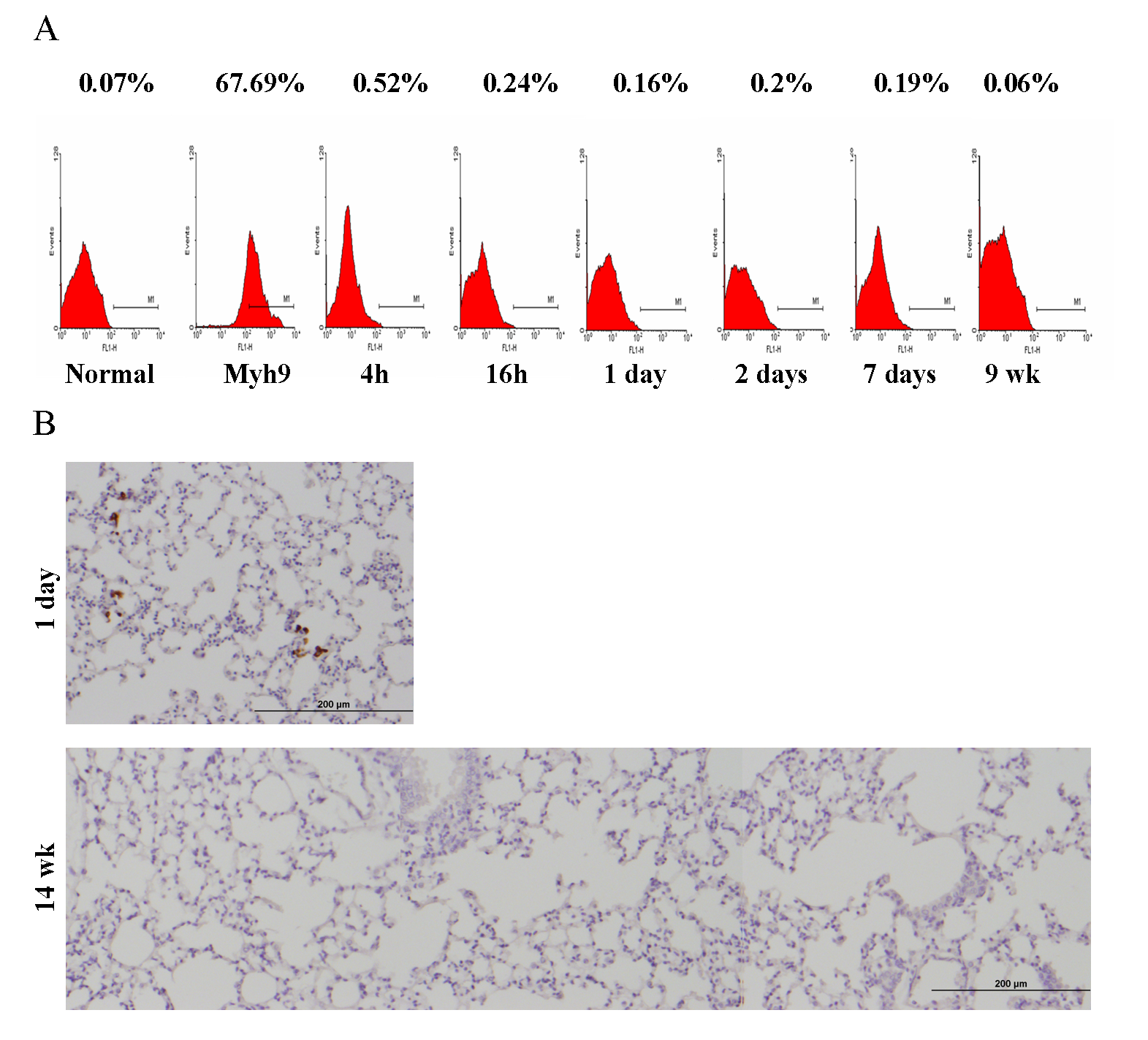

Supplement: Additional file 3: Figure S3. — Showing A quantification of GFP-positive cells in the lung after intravenous injection of MKPCs in five-sixths nephrectomized mice (y axis shows the number of cells, while the x axis (FL1-H) shows the fluorescence intensity; M1 is the area of GFP-positive cells) and B immunohistochemistry of the lung after intravenous injection of MKPCs into a mouse that underwent five-sixths nephrectomy. Few GFP positive cells were found in the lung at the first day but there were no GFP-positive cells at week 14. (TIFF 2253 kb) [file 13287_2015_241_MOESM3_ESM.tiff]

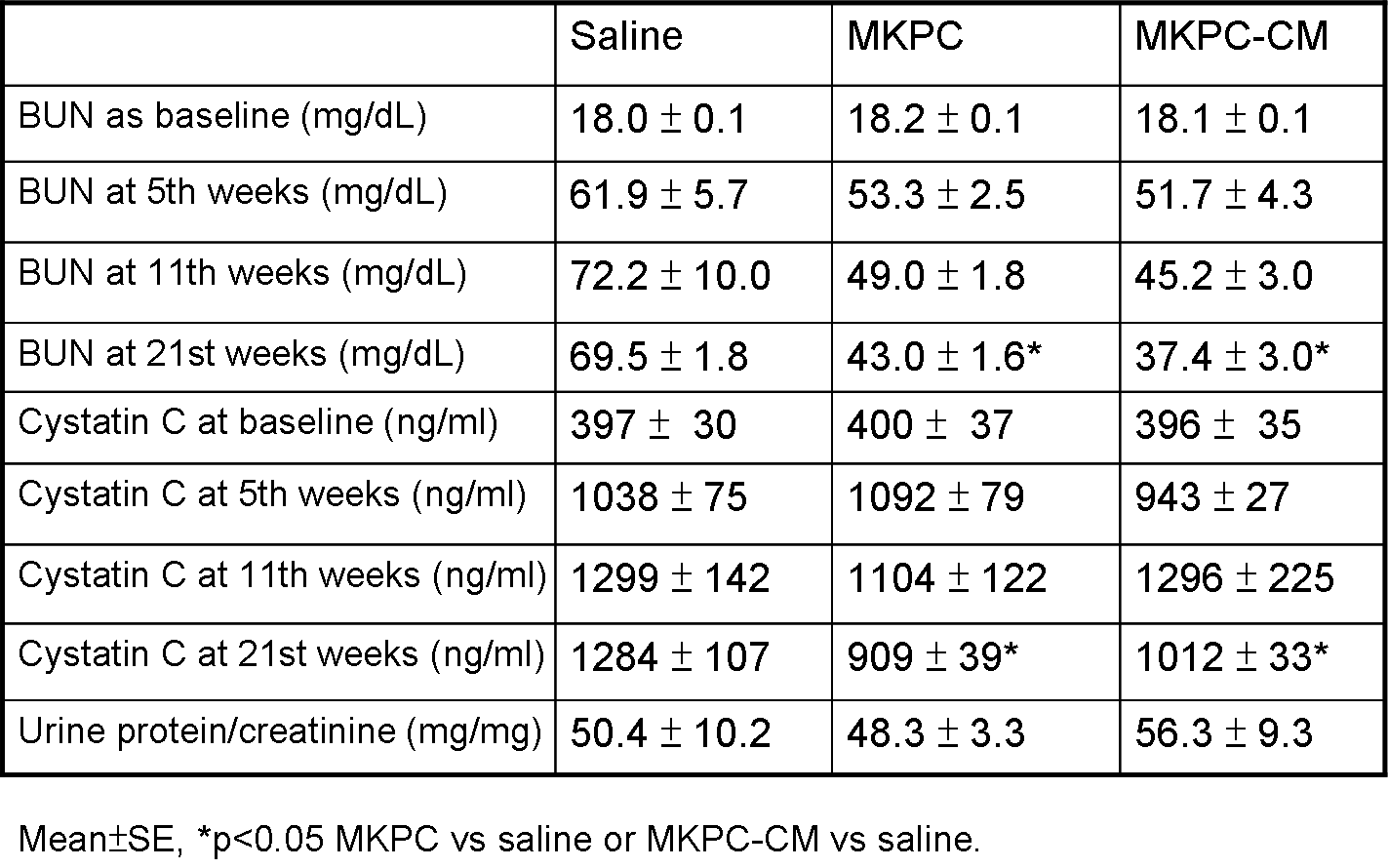

Supplement: Additional file 4: Table S1. — Showing that injections of conditioned medium derived from MKPCs improve renal function. (TIFF 144 kb) [file 13287_2015_241_MOESM4_ESM.tiff]
